# Supplementary material for: Comprehensive Molecular Dissection of Dermatophilus congolensis Genome and First Observation of tet(Z) Tetracycline Resistance
Source: Int J Mol Sci. 2021 Jul 1;22(13):7128. doi: 10.3390/ijms22137128 (PMC8267673; doi:10.3390/ijms22137128)
Supplement: Supplementary file 1 [file ijms-22-07128-s001.zip › Supplementary Table 2.pdf]

Supplementary Table 2. Virulence factor matches in Virulence Factor DataBase (VFDB) and protein identity determine through BLASTp.

Virulence Factor Class	Annotation from *D. congolensis* genomes	Gene	Species	Identity %	Accession #
Adherence	GroEL chaperonin	*groEL*	*M. tuberculosis*	72	CNF16026.1
	Streptococcal plasmin receptor/	*plr/gapA*	*Str. pneumoniae*	65	CKE63244.1
Iron uptake					
	ABC transporter	*fagA*	*M. tuberculosis*	51	CNK55089.1
	Iron siderophore ABC transporter	*fagD*	*Mycobacterium sp* LNNU 331112	38	WP_149359153.1
	Exochelin	*fxbA*	*M. smegmatis*	50	WP_029104465.1
	Exochelin	*fxuB*	*Corynebacteriales*	61	WP_064906802.1
	Iron/manganese transport	*sitA*	*Mycobacteraceae*	73	WP_019511651.1
		sitB	*Mycobacterium sp LNNU 331112*	61	WP_149359937.1
	Mycobactin (Mycobacterium)	*mbtH*	*Mycobacteriaceae*	64	WP_046283878.1
	Antranilate synthase component 1	*mbtl*	*M. sp* LNNU 331112	57	WP_149360889.1
	ABC transporter	*irtA*	*M. sp.* OK889	45	TDR65420.1
Regulation					
	Diphtheria toxin repressor DtxR	*IdeR*	*M. tuberculosis*	54	CNI59779.1
	(p)ppGpp synthesis and hydrolysis	*relA*	*M. tuberculosis*	53	CNF85191.1
	Response regulator transcription factor	*(devR/dosR)*	*Mycobacterium sp.*	52	SBS74131.1
	Response regulator transcription factor	*phoP*	*M. tuberculosis*	66	CNE26216.1
		phosR	*M. pseudoshottsii* JCM 15466	43	GAQ32061.1
	Response regulator transcription factor	*(regX3)*	*M. tuberculosis*	74	CNE05134.1
	RNA polymerase Sigma factor	*sigA*	*M. tuberculosis*	90	CNI09155.1
	RNA polymerase Sigma factor E	*sigE*	*M. tuberculosis*	72	CND44553.1
	RNA polymerase Sigma H (sigma-24)	*sigH*	*M. tuberculosis*	74	CNG47707.1
	WhiB family transcription regulator 3	*whib3*	*Mycobacterium sp.* 1245111.1	61	WP_067342063.1
Toxin					
	Phospholipase D	*pld*	*C. ulcerans*	59	WP_029975046.1
Acid resistance					
	Urease	*ureB*	*M. tuberculosis*	61	WP_057332578.1
	Urease	*ureG*	*Mycobacteria sp.* NAZ190054	64	WP_067957749.1
Amino acid and purine metabolism					
	Diaminopimelate decarboxylase	*lysA*	*M. leprae*	52	Q50140.2
	Purine synthesis	*purC*	*M. tuberculosis*	54	CNJ22485.1
	Tryptophan synthesis	*trpD*	*M. leprae*	52	WP_010907993.1
	type I glutamate ligase	*glnA1*	*M. leprae*	61	WP_010908444.1
Anti-apoptosis factor					
	NuoG(Mycobacterium)	*nuoG*	*M. avium*	61	WP_084050036.1
Antiphagocytosis					
	Nucleotide sugar dehydrogenase	*fgd*	*M. tuberculosis*	68	REN02080.1
		gnd	*Mycobacterium sp.* 3519A	60	WP_101946767.1
Cell surface components					
	Trehalose-recycling ABC transporter sugA	*sugA*	*M. tuberculosis*	55	CND65206.1
	Carbohydrate ABC transpoter permease	*sugB*	*M. tuberculosis*	63	CND65175.1
	Trehalose import ATP-binding protein	*sugC*	*M. tuberculosis*	62	CND65145.1
Copper uptake					
	Copper exporter	*ctpV*	*M. sp* D16Q16	52	WP_139360043.1
Endotoxin					
	O-antigen biosynthesis aminotransferase	*bplF*	*B. broncjseptica*	53	WP_033453820.1
Immune evasion					

	Capsule (Staphylococcus)	*cap5G*	*S. aureus*	51	HAR5208460.1
	Exopolysaccharide (Haemophilus)	*pgi*	*M. noviomagense*	61	WP_083087194.1
	polysaccharide biosynthesis protein	*gna*	*Acinetobacter sp.* YH12025	56	WP_180175575.1
Motility and export apparatus					
	Flagellar	*fliP*	*Austwickia sp.*	67	QQS02820.1
Others					
	O-antigen (Yersinia)				
	Glucose-1-phosphate cytidylyltransferase	*ddhA*	*Y. enterocolitica*	63	WP_174847827.1
	GDP-L-fucose synthase	*wcaG*	*Y. pseudotuberculosis*	54	AKA21002.1
Phagosome arresting					
	Nucleoside diphosphate kinase (Mycobacterium)	*ndk*	*M. gordonae*	61	WP_180914471.1
Protease					
	Proteasome-associated proteins	*mpa*	*M. tuberculosis*	53	CNF79738.1
	Zn++ metallophrotease (peptide m13)	*Zmp1*	*M. Leprae*	53	SRX92647.1
Secretion system					
	Mxi-Spa TTSS effectors controlled by MxiE (Shigella)	*lhgO*	*Mycobacterium sp.* MS1601	54	WP_083736669.1
	T6SS-II	*clpB*	*M. tuberculosis*	68	CNH94644.1
Stress adaptation					
	Iron-co-factored SOD (Mycobacterium)	*sodA*	*M. avium*	64	WP_062889796.1
Nutritional virulence					
	Pyrimidine biosynthesis	*carB*	*M. tuberculosis*	74	CNE60285.1
