# Supplementary material for: Comprehensive Molecular Dissection of Dermatophilus congolensis Genome and First Observation of tet(Z) Tetracycline Resistance
Source: Int J Mol Sci. 2021 Jul 1;22(13):7128. doi: 10.3390/ijms22137128 (PMC8267673; doi:10.3390/ijms22137128)
Supplement: Supplementary file 1 [file ijms-22-07128-s001.zip › Supplementary Table 3.pdf]

Supplementary Table 3. Gene clusters of secondary metabolites found in 40 genomes of *D. congolensis* detected by antiSMASH software 5.0.

Genome	Secondary metabolite gene cluster			
	NRPS	Bacteriocin	Siderophore	Terpene
BTSK1	2	1	-	-
BTSK2	2	1	-	-
BTSK3	2	-	-	-
BTSK4	2	-	1	-
BTSK5	2	-	-	-
BTSK6	2	-	-	-
BTSK7	2	1	-	-
BTSK8	2	1	-	-
BTSK9	2	-	-	-
BTSK10	2	-	-	-
BTSK11	2	-	-	-
BTSK12	2	-	-	-
BTSK13	2	-	1	-
BTSK14	2	-	-	-
BTSK15	2	-	1	-
BTSK16	2	-	1	-
BTSK17	2	1	-	-
BTSK18	2	-	1	-
BTSK19	2	-	1	-
BTSK20	2	-	-	-
BTSK21	2	1	-	-
BTSK22	2	-	-	1
BTSK23	2	-	1	-
BTSK24	1	-	1	-
BTSK25	2	-	-	-
BTSK26	2	-	1	-
BTSK27	2	1	-	-
BTSK28	2	-	-	-
BTSK29	2	-	1	-
BTSK30	2	-	1	-
BTSK31	2	1	-	-
BTSK32	2	-	-	-
BTSK33	2	-	1	-
BTSK34	2	1	-	1
BTSK35	2	-	-	-
BTSK36	2	-	-	1
BTSK37	2	-	1	1
BTSK38	2	-	-	-
BTSK39	2	-	-	-
BTSK40	2	-	-	-
