# Supplementary figures and images for: Comprehensive Molecular Dissection of Dermatophilus congolensis Genome and First Observation of tet(Z) Tetracycline Resistance
Source: Int J Mol Sci. 2021 Jul 1;22(13):7128. doi: 10.3390/ijms22137128 (PMC8267673; doi:10.3390/ijms22137128)

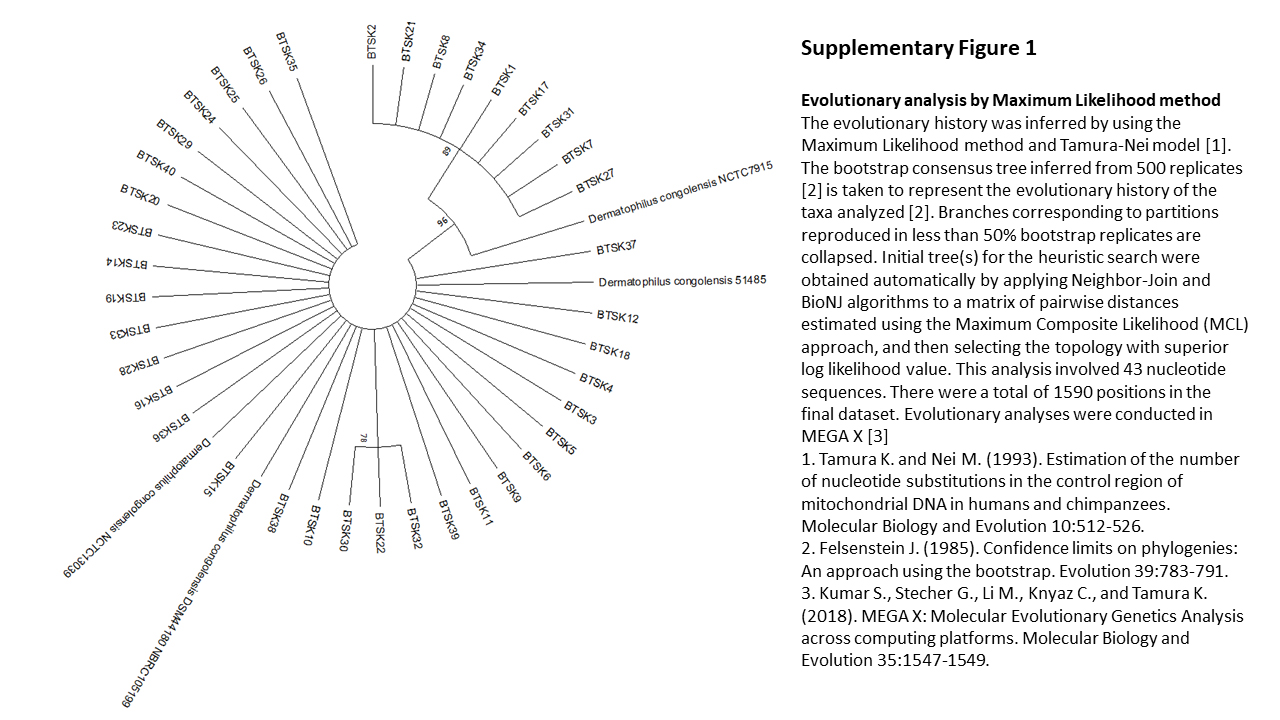

Supplement: Supplementary file 1 [file ijms-22-07128-s001.zip › Supplementary Figure 1.jpg]

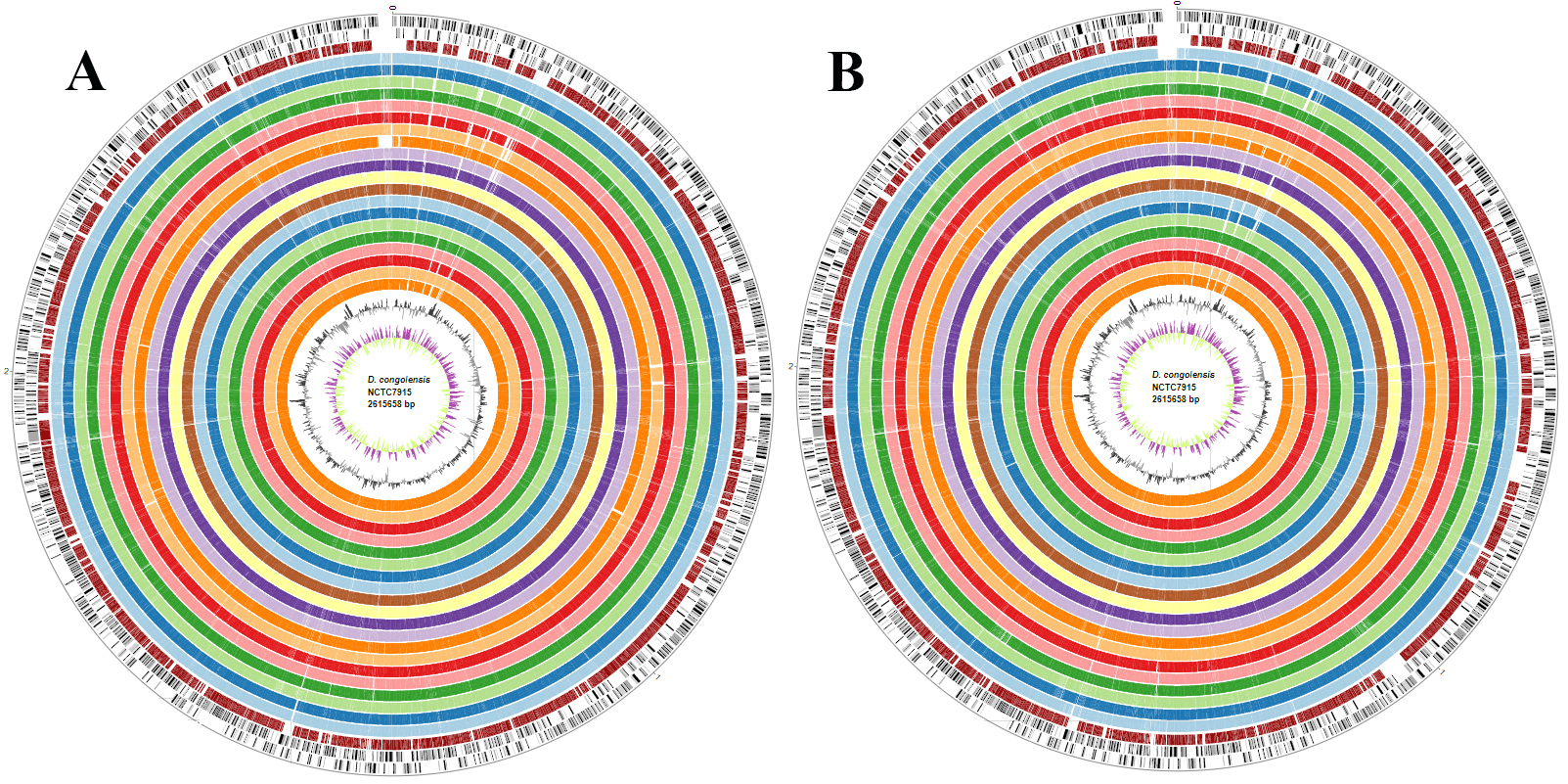

Supplement: Supplementary file 1 [file ijms-22-07128-s001.zip › Supplementary Figure 2.jpg]

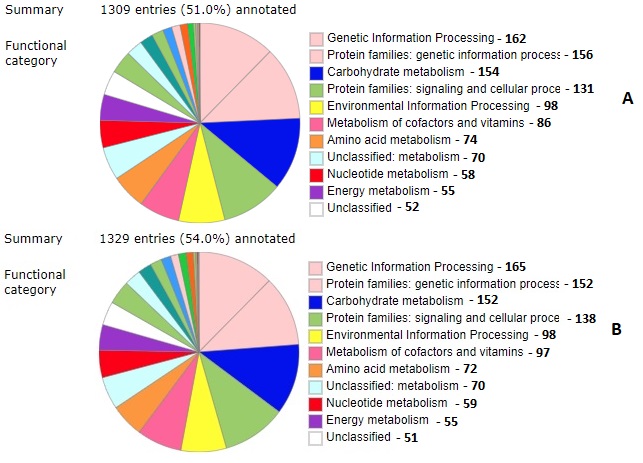

Supplement: Supplementary file 1 [file ijms-22-07128-s001.zip › Supplementary Figure 3.jpg]
